# Role of Vitamin D in Osteoarthritis: Molecular, Cellular, and Clinical Perspectives

**DOI:** 10.1155/2015/383918

**Published:** 2015-07-02

**Authors:** Thomas Mabey, Sittisak Honsawek

**Affiliations:** ^1^Department of Biochemistry, Faculty of Medicine, Chulalongkorn University, King Chulalongkorn Memorial Hospital, Thai Red Cross Society, Bangkok 10330, Thailand; ^2^Department of Orthopaedics, Faculty of Medicine, Chulalongkorn University, King Chulalongkorn Memorial Hospital, Thai Red Cross Society, Bangkok 10330, Thailand

## Abstract

Osteoarthritis is a debilitating and degenerative disease which affects millions of people worldwide. The causes and mechanisms of osteoarthritis remain to be fully understood. Vitamin D has been hypothesised to play essential roles in a number of diseases including osteoarthritis. Many cell types within osteoarthritic joints appear to experience negative effects often at increased sensitivity to vitamin D. These findings contrast clinical research which has identified vitamin D deficiency to have a worryingly high prevalence among osteoarthritis patients. Randomised-controlled trial is considered to be the most rigorous way of determining the effects of vitamin D supplementation on the development of osteoarthritis. Studies into the effects of low vitamin D levels on pain and joint function have to date yielded controversial results. Due to the apparent conflicting effects of vitamin D in knee osteoarthritis, further research is required to fully elucidate its role in the development and progression of the disease as well as assess the efficacy and safety of vitamin D supplementation as a therapeutic strategy.

## 1. Introduction 

Osteoarthritis (OA) is a progressive and degenerative joint disease. Commonly affecting weight bearing synovial joints, OA is characterised by the degradation and loss of articular cartilage, abnormal subchondral bone growth and remodeling, and, in early stages, inflammation of the synovium. The complexity of OA has hindered attempts to understand its aetiology which still remains elusive. There are, however, a range of risk factors known to associate with OA including age, gender, obesity, previous joint trauma, and genetics [1]. Historically, it was assumed that only cartilage contributed to the progression of OA, but all tissues within the joint structure are now known to be involved. In osteoarthritic knees, the most common location of OA, the subchondral tibial and femoral bones play central roles in the pathology of joint degeneration. Subchondral bone sclerosis, joint space narrowing, osteophyte formation, and loss of bone contours are evaluated using the Kellgren-Lawrence grading system for assessment of osteoarthritis [2] and reflect the severity of joint changes via radiography.

Vitamin D is a steroidal hormone that has many diverse biological actions in a number of target tissues. The primary functions of vitamin D are calcium homeostasis and regulation of bone metabolism; however, the full extent of vitamin D's biological action remains to be determined with a wide range of effects on different cell and tissue types being reported. Acting via the vitamin D receptor (VDR), vitamin D regulates circulating calcium and phosphate homeostasis through altering kidney reabsorption and intestinal absorption [3]. Parathyroid hormone (PTH) and fibroblast growth factor- (FGF-) 23, a bone-derived phosphaturic hormone produced in the presence of active vitamin D [4], are also major players involved in the maintenance of these circulating ion levels. PTH is secreted by the parathyroid glands in response to low calcium levels and acts to stimulate active vitamin D synthesis. This is achieved by inducing the release of calcium into the circulation via increased bone turnover to prevent hypocalcaemia [5]. With such a potent effect on bone, vitamin D has been investigated as to its role in OA. To date, numerous studies have shown its involvement and association with many aspects of the disease. Here we aim to review the current understanding of vitamin D and the roles it plays in osteoarthritis.

## 2. Vitamin D Forms and Biosynthesis 

**Figure 1:**
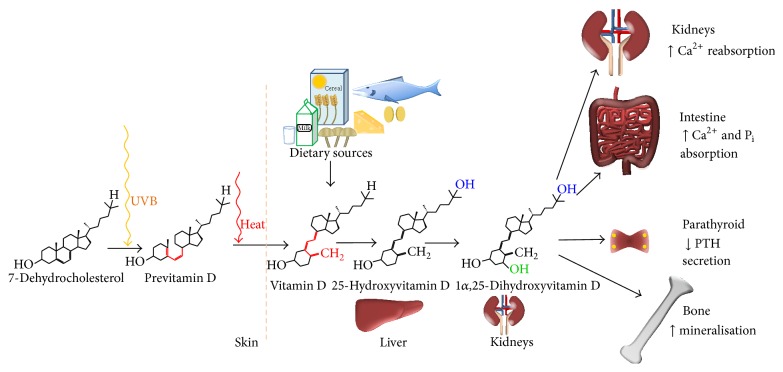
The biosynthesis of vitamin D and the major effects 1*α*,25(OH)_2_D_3_ has on different organs. Synthesis begins in the skin before activation in the liver and kidneys. PTH: parathyroid hormone, Ca: calcium, and P_i_: inorganic phosphate.

Vitamin D has two main forms, D_2_ and D_3_. Vitamin D_2_, also known as ergocalciferol, is produced predominantly by plants and fungi and forms part of the dietary intake of vitamin D. Vitamin D_3_ is the endogenous form produced by humans. Biosynthesis begins with the photoisomerisation of 7-dehydrocholesterol (DHC) by ultraviolet B (UVB) radiation to form previtamin D in cutaneous tissue (Figure 1) [6]. Previtamin D then undergoes thermal-dependent isomerization to form vitamin D_3_ [cholecalciferol] [7]. The lipophilic cholecalciferol is biologically inactive and requires two sequential hydroxylations to reach its most active form. For this to occur, cholecalciferol preferentially binds to vitamin D binding protein (DBP) and is transported to the liver wherein hydroxylation of the 25-position carbon follows; the product of this reaction is 25-hydroxyvitamin D_3_ [calcidiol; 25(OH)D_3_]. It is this 25(OH)D_3_ form which is usually measured in serum to determine vitamin D levels in patients. An additional hydroxy group is added to the carbon at the 1 position through further hydroxylation which occurs primarily in the proximal tubule of the kidneys, but also in macrophages [8], osteoblasts [9], and osteoclasts [10]* inter alia*. The result is the most biologically active form of vitamin D, 1*α*,25-dihydroxyvitamin D_3_ [calcitriol; 1*α*,25(OH)_2_D_3_]. Names and abbreviations of vitamin D_3_ are shown in Table 1.

## 3. Vitamin D Receptor 

The vitamin D receptor (VDR), through which 1*α*,25(OH)_2_D_3_ acts, is a nuclear transcription factor which regulates gene expression in various cell types. Upon binding with vitamin D, VDR forms a heterodimer with the retinoid X receptor (RXR). The resultant complex recognises and binds vitamin D response elements (VDREs) which consists of 2 hexameric motifs separated by a 3-base-pair spacer. Genome-wide studies have identified between 2,000 and 8,000 VDREs depending on cell type [30, 31].

Due to its crucial role in vitamin D signalling, the* VDR* gene and its corresponding protein have been subject to much investigation in many diseases including osteoarthritis. In particular, genetic polymorphisms in the* VDR* gene and its surrounding regulatory sites, some of which can be located at large distances from the open reading frame, have been investigated using restriction fragment length polymorphism (RFLP). The technique employs restriction enzymes to cut DNA at specific sequence sites. The polymorphisms detected in the DNA, the fragments of which are analysed using gel electrophoresis and Southern blotting, include single nucleotide polymorphisms (SNPs) and insertion/deletion (INDEL) polymorphisms as they can alter the cleavage sites of the endoribonuclease and are therefore identifiable. In the* VDR* gene, a number of polymorphisms have been identified and studied named* EcoR*V,* Tru9*I,* Fok*I,* Bsm*I,* Apa*I, and* Taq*I after the restriction enzymes used to identify them [32]. The latter four have been at the centre of investigations for possible involvement in OA.* Bsm*I and* Apa*I are both located in intron 8, and* Taq*I is found in exon 9 [33]. These SNPs are considered synonymous due to the fact that they do not change the amino acid residue translated for in the final protein product. Conversely,* Fok*I is considered nonsynonymous as the T/C polymorphism ultimately changes the amino acid sequence and in doing so changes the start codon location meaning 2 protein forms of VDR exist, one of which is 3 amino acids longer than the other [32]. The reader is directed to a review by Uitterlinden et al. [32] for further details on these polymorphisms.

Despite the functional effects of these polymorphisms remaining elusive, four polymorphisms have been of particular interest in investigations in association studies with OA. It has been discovered that genetic polymorphisms may be associated with an increased susceptibility of symmetrical hand OA in Finnish females [34]. Additionally, a study in 1997 of postmenopausal women with knee OA found a possible association between a* Taq*I polymorphism and an increased risk of OA [35]. In 2009, Lee et al. [36] performed a meta-analysis of 10 studies (1,591 OA patients and 1,781 controls) into the association of the* Taq*I,* Bsm*I, and* Apa*I polymorphisms with osteoarthritis. The studies analysed had investigated a range of the OA sites, including knee, lumbar spine, hand, and hip. Overall, it was found that there was no evidence to support an association between any of the SNPs and a susceptibility to OA; additionally, no association was found when studies were grouped into ethnicities (European or Asian). This conflicts with a more recent meta-analysis in which it was found that a small but statistically significant association exists between the* Apa*I polymorphism and the susceptibility to OA in Asian populations, an association not observed in Europeans [37]. This analysis included 9 of the previous 10 studies in addition to 3 new reports with a total of 2,104 OA patients and 2,939 controls. A possible association was discovered between the* Fok*I polymorphism and OA, but the small number of studies limits the power of this analysis.

Whilst it appears that SNPs in the* VDR* gene show little if any association with knee OA in large groups, analysis of subgroups and ethnicities has shown possible links. Investigations into relationships between SNPs and different aspects of OA, for instance, joint space narrowing (JSN), osteophyte formation, or inflammation, will continue to be valuable. With the advent of next-generation sequencing, genome-wide association studies (GWASs) offer a global genome perspective not attainable with the inherently biased candidate gene association studies. Two such studies performed on large scale population sizes failed to identify* VDR* gene polymorphisms to have a significant association with OA [38, 39]. Moreover, a meta-analysis of 9 GWASs which included 5,636 knee OA patients and 16,972 controls showed no significant association between SNPs in the* VDR* gene and OA [40]. Combinations of SNPs from* VDR* and other OA-related genes, as opposed to assessing the involvements of a single polymorphism, may yield a more powerful insight into the genetic aspect of OA. However, the number of polymorphisms in the genome and the possible number of combinations present a challenge due to the sheer volume of data and the ability to analyze it.

## 4. Vitamin D and Bone 

**Figure 2:**
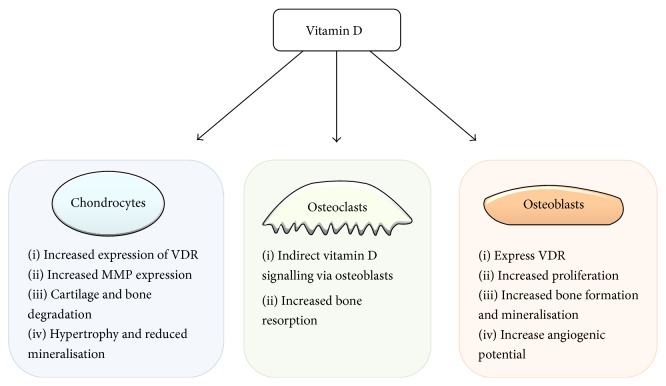
The effects of vitamin D on several different cell types in osteoarthritis including chondrocytes, osteoclasts, and osteoblasts. Vitamin D has a range of effects on cell types within osteoarthritis affected joints. Vitamin D acts through the vitamin D receptor and acts to alter gene expression which results in the phenotypic changes summarised here. Osteoclasts appear to respond indirectly to vitamin D signalling via osteoblasts and RANKL signalling. VDR: vitamin D receptor; MMP: matrix metalloproteinase.

Contrary to historic opinion, osteoarthritis affects all tissues within the joint; the major tissue types include articular cartilage, subchondral bone, and the synovial membrane lining the diseased joint. Alterations in the physiological processes occurring in these tissues ultimately lead to the progression of OA. There are two main cell types in the subchondral bone which are of particular interest in OA: osteoblasts and osteoclasts. Changes in normal cellular activities cause aberrant bone remodeling, sclerosis, and osteophyte formation. Vitamin D is known to play a crucial role in the behaviour of metabolic processes in bone on which it has a range of effects in the pathophysiology of OA. A number of recent articles have reviewed the effects and roles of vitamin D in bone cells in detail [41–43]. Here, we look specifically at the changes in cellular behaviour in osteoarthritis (Figure 2).

### 4.1. Osteoblasts

Osteoblasts are single-nuclei cells derived from mesenchymal stem cells, a process which can be stimulated by 1*α*,25(OH)_2_D_3_ [44] and whose primary functions are bone formation and mineralisation. Osteoblasts, particularly immature cells, express VDR [45, 46]; this shows osteoblasts as key targets for vitamin D signalling. The phenotypic expressions of osteoblasts in OA are altered [47]. A study by Corrado et al. [48] found a number of interesting results when they investigated osteoblasts isolated from hip OA patients as well as healthy controls and osteoporosis patients. OA osteoblasts expressed a significantly lower receptor activator of nuclear factor-*κ* B ligand (RANKL)/osteoprotegerin (OPG) ratio compared to healthy and osteoporotic cells and OPG expression in OA osteoblasts was significantly higher than controls and osteoporosis patients. Interestingly, these findings conflict with the results of Giner et al. [49] who found OPG secretions to be higher in osteoporotic osteoblasts compared to OA. RANKL and its decoy receptor OPG are regulators of osteoclastogenesis and thus bone resorption. Vitamin D, specifically 1*α*,25(OH)_2_D_3_, is known to stimulate osteoblastic bone mineralisation through the activation of nuclear VDR [44]. Proliferation of OA osteoblasts was significantly increased following vitamin D treatment compared to that of healthy osteoblasts [48]. Additionally, osteocalcin and alkaline phosphatase (two proteins involved in the formation and mineralisation of bone) production were both significantly higher in OA osteoblasts compared to healthy and osteoporotic ones. The productions were significantly increased following vitamin D treatment [48]. This work supports Hilal and colleagues' investigation which found that 1*α*,25(OH)_2_D_3_ induced a significantly higher production of osteocalcin in OA osteoblast-like cells compared to controls [47]. Taken together, these findings suggest that OA osteoblasts have increased vitamin D-induced bone formation activity which could help to explain subchondral sclerosis and osteophyte formation, both of which are characteristics of advanced OA.

The Dickkopf (DKK) protein factor family is involved in the activation and inhibition of Wingless (Wnt) signalling induced bone formation through osteoblast proliferation and activity which in turn regulates bone remodeling [48, 50]. DKK-1 expression was significantly lower in OA osteoblasts compared to healthy and osteoporotic cells; following vitamin D treatment, the expressions levels decreased significantly [48]. Conversely, DKK-2 expression was significantly higher in OA osteoblasts and was further increased with the addition of vitamin D [48]. These findings indicate the involvement of vitamin D in the cellular development of osteoblasts and support our own study in which significantly lower DKK-1 levels were found in plasma samples of OA patients compared with healthy controls [51].

An important aspect in the pathophysiology of OA is angiogenesis. The development and extension of vascular networks have been observed in the synovium, menisci, pannus, osteophytes, and osteochondral junction within affected OA joints [52]. Whilst an increase in blood supply to the tissues within an OA joint may be beneficial by supplying nutrients for cartilage repair, this growth is associated with the increase in pain through the extension of sensory nerves [53] and the loss of structural integrity of cartilage. Vascular endothelial growth factor (VEGF) is a potent angiogenic cytokine which plays an important role in OA. Our laboratory has shown both local and systemic levels to be associated with the severity of OA [54]. The expression of VEGF is regulated in part by 1*α*,25(OH)_2_D_3_ in osteoarthritic osteoblasts [55]. Additionally, the angiogenic potential of the culture medium taken from OA osteoblasts was significantly increased following vitamin D treatment* in vivo* [55]. These observations support the idea that osteoblasts may regulate angiogenesis in subchondral bone and therefore may link vitamin D with the development and progression of OA.

### 4.2. Osteoclasts

Osteoclasts are large multinuclear cells with characteristic ruffled edges and sealing zones. Formed from the fusion of multiple preosteoclast cells, these specialised macrophages' primary function is the resorption of mineralised bone. This occurs in the resorption lacuna under acidic conditions by proteases such as cathepsin K, matrix metalloproteinase- (MMP-) 9, and tartrate-resistant acid phosphatase. Whilst high doses of vitamin D have been shown to increase bone resorption [56, 57], there is limited evidence to suggest that vitamin D acts directly on osteoclasts due to the lack of VDR expression, though reports are conflicting and dependent on techniques used [45, 58–60]. However, vitamin D is thought to act indirectly through osteoblastic activity and the activation of the aforementioned RANKL signalling.

### 4.3. Chondrocytes

Chondrocytes are the cell type responsible for the production and maintenance of the extracellular matrix (ECM) in cartilage. In OA, imbalances in the anabolic and catabolic processes result in the destruction and loss of articular cartilage ultimately leading to the progression of OA. Articular cartilage in the knee is typically avascular and receives nutrients and growth factors primarily from the synovial fluid. Additionally, chondrocytes exist in a relatively low cell density and as a result ECM production and repair is a slow process. Hypertrophic and proliferating chondrocytes express VDR [45, 46] and osteoarthritic chondrocytes more so than healthy cells [61, 62]. Additionally, the presence of VDR expression is associated with MMP expression, specifically MMP-1, MMP-3, and MMP-9. MMPs enzymatically degrade bone and cartilage, which is disadvantageous in OA. Chondrocytes have been shown to directly regulate osteoclastogenesis through VDR signalling leading to the induction of RANKL expression [63]. Furthermore, 1*α*,25(OH)_2_D_3_ and inorganic phosphate are involved in the hypertrophy and impaired mineralisation in osteoarthritic chondrocytes by activating extracellular-signal-regulated kinase (ERK)1/2 through FGF-23 signalling [62]. From these reports, it appears that vitamin D has negative effects on OA cartilage health. Future research should aim to investigate the extent and severity of these actions and their implications on the development and progression of OA.

## 5. Vitamin D Deficiency and OA 

Vitamin D deficiency results in hypocalcemia and hypophosphatemia in addition to increase in PTH secretion. Hypovitaminosis D, generally (though not universally) defined as circulating serum 25(OH)D_3_ levels of <20 ng/mL (Table 2), is prevalent worldwide [64] and frequently found to coexist with OA, particularly in the elderly patients. 24% of advanced stage elderly OA patients in a United Kingdom study were found to have deficient vitamin D levels according to the National Diet and Nutrition Survey definition of less than 40 nmol/L (~16 ng/mL) [65]. In a 2010 study in Ireland of rheumatology outpatients, 70% were found to be vitamin D deficient (<21 ng/mL) and 26% were severely deficient (<12 ng/mL) [66]. The study also noted 62% of OA patients suffered from hypovitaminosis D and 13% were severely affected. Additionally, low vitamin D levels have also been associated with radiographic hip OA [11].

It has been shown that the capacity of human skin to produce vitamin D decreases in old age [67]. Furthermore, vitamin D levels are associated with other known risk factors of OA including body mass index (BMI), age, very heavy manual labour, and exercise [68]. Research has been focused on investigating possible relationships between vitamin D levels and aspects of OA, as summarised in Table 3.

### 5.1. Development and Progression

Whether vitamin D deficiency increases the risk of developing OA remains to be answered. Conflicting results have been reported to date. A 22-year follow-up study of 805 Finns found that serum 25(OH)D_3_ levels were not associated with the incidence of either hip or knee OA [68]. This study supports an earlier report in which low vitamin D was found not to increase the risk of developing knee OA [12]. Furthermore, a large cohort study of 5,274 OA-free participants showed that low serum 25(OH)D_3_ levels were not associated with an increased risk of developing hip or knee OA over a 10-year period [13]. However, contrary to these studies, lower serum vitamin D has been shown to be associated with OA. As part of the Osteoporosis Fractures in Men Study in the United States, an investigation found a high prevalence of vitamin D insufficiency or deficiency in hip OA patients and found that these patients were twice as likely to have hip OA [14]. In Egypt, research into newly diagnosed postmenopausal women found lower serum 25(OH)D_3_ was associated with knee OA when compared to healthy males [15]. Furthermore, an Iranian study observed a positive association between serum 25(OH)D_3_ and knee OA in patients under 60 years of age and noted a stronger association in younger participants [16]. Additionally, in patients with low BMD, low vitamin D status was found to be associated with an increased incidence of radiographic knee OA [17]. A systematic review performed by Cao et al. examined the associations between serum 25(OH)D_3_ and OA [69]. They found through analysing 15 studies that there exists strong evidence for an association between 25(OH)D_3_ and cartilage loss in knee joints; the authors also observed moderate evidence to support a positive association between low levels of vitamin D and radiographic knee OA. Low serum and low dietary vitamin D have been shown to be associated with the progression of knee OA [12, 17]. Moreover, as part of the Tasmanian Older Adult Cohort Study, Ding et al. found both vitamin D levels and sunlight exposure to be associated with decreased knee cartilage loss [18]. However, Felson et al. detailed two longitudinal studies with a total study population of 1,203 individuals. They found that there was no association between low vitamin D and structural worsening of affected joints (joint space narrowing by radiography and cartilage loss by magnetic resonance imaging) [19].

### 5.2. Pain and Function

Pain is a major symptom of OA, particularly in the knee. Pain is thought to be caused by a number of reasons including inflammation of the synovial membrane and the growth of sensory nerves through the subchondral bone into the articular cartilage [53]. Studies have shown conflicting results as to whether vitamin D is associated with pain in OA. In a 2-year randomised control study in which symptomatic knee OA patients were given oral doses of cholecalciferol to raise circulating levels to at least 36 ng/mL or a placebo, there was no reduction in the Western Ontario and McMaster Universities (WOMAC) knee pain scores or cartilage loss [29]. Additionally, 787 members of the Hertfordshire Cohort Study in the United Kingdom took part in a cross-sectional study that found no association between vitamin D levels and radiographic knee OA but did suggest a significant association between vitamin D and knee pain [20]. However, a longitudinal population-based cohort study of 769 participants found that moderate vitamin D deficiency (serum 25(OH)D_3_ = 25 nmol/L) was found to predict knee OA WOMAC pain scores over 5 years. Additionally, a similar trend was observed in hip pain scores, though this association did not reach statistical significance [21]. Furthermore, a double-blind parallel, placebo-controlled pilot trial of 103 knee OA patients found patients given vitamin D oral supplements (60,000 IU per day for 10 days followed by 60,000 IU once a month for 12 months) had slightly higher pain and functional scores compared to those receiving placebos after one-year follow-up [28]. However, seasonal vitamin D levels have been found not to be correlated with WOMAC scores in knee OA patients, nor did they associate with rheumatoid arthritis or ankylosing spondylitis disease activity [22].

Joint function is a useful indicator of OA as it reflects the socioeconomic effects of OA as well as the patients' quality of life. Vitamin D deficiency has been associated with preoperative Knee Society scores of functionality in OA patients undergoing arthroplasty surgery [65]. The same study found lower postoperative scores in vitamin D deficient patients than vitamin D sufficient patients though this did not reach statistical significance. A study of knee OA patients in Kuwait found that 93% were vitamin D deficient; however, neither X-ray grading nor functionality was associated with hypovitaminosis D [23]. Vitamin D levels have been shown to predict the outcomes of OA patients undergoing total hip replacement. A positive correlation exists between both preoperative and postoperative Harris hip scores and plasma 25(OH)D_3_ levels [70]. The research presented to date is conflicting as to the effects low vitamin D levels have on the functional aspects of knee OA.

### 5.3. Bone Mineral Density

Bone mineral density (BMD) has also been investigated due to the crucial involvement vitamin D has in bone metabolism. A study of 228 primary knee OA patients found that there was a significant positive association between the BMD of the femoral neck and serum 25(OH)D_3_ which was independent of age, sex, BMI, knee pain, level of physical activity, and disease severity [24]. Moreover, a 2-year longitudinal study containing 1,122 males, as part of a population-based study in Australia, found that low serum 25(OH)D_3_ put men at increased risk of low BMD [71]. Bone mineral density was seen to increase following a 16-week weight loss program in which 175 obese knee OA patients received formula products which were enriched with vitamin D. Vitamin D levels rose significantly during the study as PTH levels decreased [25]. Further, a study of 56 hip OA patients who had undergone primary total hip arthroplasty found that BMD loss was prevented in the lumbar spine by vitamin D analogues alfacalcidol (1*α*(OH)D_3_) and alendronate compared with no-treatment controls [72]. However, Breijawi et al. discovered no association or correlation between serum vitamin D levels and BMD of either lumbar spine or proximal femur sites, though they did note a high prevalence of hypovitaminosis D in their hip and knee OA patients [73]. Moreover, in a twin study of 1,644 females, vitamin D levels were not lower in OA patients after adjusting for BMI and age nor were there any significant associations with changes to subchondral bone or bone remodelling [26]. Despite some conflicting results, there appears to be an association between low circulating vitamin D and low BMD in OA patients. A decrease in BMD is associated with progressive cartilage loss in knee OA [74, 75] and so reflects a wide view of the disease in different tissues. Osteoblast activity is increased in OA and so it would be expected that higher BMD would follow. However, BMD is usually measured in the lumbar spine or femoral neck, sights often not associated with OA in the patients under investigations. Sclerosis of subchondral bone occurring in affected joint is characteristic of OA. It could be hypothesised that the effects of vitamin D are felt differently in different tissues. Studies investigating localised expression of VDR and the resultant effects of vitamin D signalling are needed.

The results of the studies thus far bespeak an unclear depiction of the associations vitamin D has with OA. Whilst there appears to be substantial evidence for low vitamin D levels being connected with the incidence of OA and low BMD, these results are not without their controversy. There currently exists insufficient evidence to draw reasonable conclusions with respect to pain and functional changes depending on circulating vitamin D concentrations. The controversial results presented herein may owe their differences to a number of factors. Different techniques and laboratory procedures have varying degrees of accuracy in determining vitamin D levels. Study populations in different geographical locations as well as different ethnic backgrounds could also explain their opposing conclusions. Hypovitaminosis D research could be further hindered by the fact that the limit at which circulating 25(OH)D_3_ concentrations become deficient has yet to be universally defined. Research has attempted to make such a definition using a range of techniques and it appears at least 50 nmol/L (~20 ng/mL) of serum 25(OH)D_3_ is necessary to normalise PTH levels and for optimal bone cell function ([76] and the references therein). Nonetheless, assessing a range of serum levels will aid in the determination of low or varying levels of vitamin D and their association with OA. To fully determine the relationship between vitamin D, as well as hypovitaminosis D, and the development and progression of OA, large scale longitudinal studies conducted at multiple centres at different geographical latitudes and climates, along with including individuals with various skin pigmentations and of different ethnicities, are required. In conjunction with further studies, large scale meta-analyses will yield more powerful interpretations of the existing findings and help to elucidate any associations low vitamin D has with OA.

### 5.4. Body Composition

BMI is a strong risk factor associated with OA and particularly knee OA where a 5 kg/m^2^ increase in BMI is associated with a 35% increase in the risk of knee OA [77]. Vitamin D signalling has been observed to play important roles in adipose tissue [78]. Moreover, inverse correlations between vitamin D concentrations and BMI as well as waist circumference and regional adiposity have been observed in a range of individuals [79–81]. Interestingly, a study of 48 bariatric females undergoing gastric bypass surgery which found that obese patients have lower serum vitamin D levels than controls also suggested that the low vitamin D in OA patients was the consequence of less sun exposure rather than as a result of OA [82]. Muscle strength has been reported to play an important role in knee OA [83] as quadriceps weakness is associated with increased joint space narrowing [84, 85]. Vitamin D deficiency is associated with decreased quadriceps strength in knee OA patients [27]. However, higher thigh muscle mass does not appear to imply protection against the development or progression of knee OA [86]. From this, it is important to consider the effects of low vitamin D and body composition in knee OA. When performing and interpreting meta-analysis in the future, caution should be taken to consider the relationship vitamin D has with BMI, muscle strength, and muscle mass and the collective effects on the disease.

## 6. Increasing Vitamin D Levels in Patients

Whilst further research is required to fully understand the role of vitamin D in OA, it is apparent that vitamin D deficiency may play a role in the pathogenesis of osteoarthritis on a clinical level. However, these effects are not mirrored at the cellular level where results indicate vitamin D has detrimental effects on joint tissues. The reason behind these conflictions has yet to be elucidated and a paucity of large scale clinical trials exists to test the long-term effects of vitamin D supplementation on knee OA [28, 29].  Table 4 summarises these trials which are not without controversy and opinions remain divided as to the benefits of vitamin D supplementation. Whilst there are some overlaps, the trials hitherto are in different populations with differing overall baseline vitamin D statuses and different dosage strategies and follow-up periods. This makes drawing conclusions difficult and limited. Moreover, vitamin D appears to be implemented more so with structural changes within OA affected joints, such as cartilage loss, rather than symptomatic aspects of the disease, for instance, pain, function, and stiffness [69]. However, the clinicaltrials.gov website currently lists a number of trials which are ongoing that aim to shed light on the safety and efficacy of vitamin D supplementation in OA and possible benefits against the disease development and progression. Additionally, little work has been completed to determine the optimal dose of supplement for maximum effect with minimal negative effects. Consequently, it would be premature to pass judgment on the use of such treatment methods in the context of knee OA. Instead, more research into the mechanisms in which vitamin D deficiency is linked to disease development and progression, as well as its use as a preventative or mitigating treatment, would be useful.

## 7. Concluding Remarks 

Vitamin D plays a crucial role in bone metabolism. It has a range of effects on various cell types within joints which have been shown to be altered on osteoarthritis. Subchondral bone sclerosis is a hallmark of OA, but so too is bone remodeling which is associated with loss of bone density, increased porosity, and transient bone loss, a paradox explored by Burr and Gallant [87]. It could be postulated that vitamin D, with its conflicting actions on bone growth, may be involved in the changes in bone behaviours at different stages of OA progression. Further studies are required to settle the debate as to the role of vitamin D deficiency in the development and progression of OA. Moreover, the relationships between low vitamin D levels and pain and joint function have yet to be fully elucidated.

## Figures and Tables

**Table 1:** Names and abbreviations of the forms of vitamin D_3_.

Name	Alternative name	Abbreviation	Activity
Vitamin D_3_	Cholecalciferol	—	Inactive
25-Hydroxyvitamin D_3_	Calcidiol	25(OH)D_3_	Active
1*α*,25-Dihydroxyvitamin D_3_	Calcitriol	1*α*,25(OH)_2_D_3_	Most active

**Table 2:** Common classifications of circulating vitamin D levels.

Classification	25(OH)D concentration	
Sufficient	>20 ng/mL	>49.92 nmol/L
Insufficient	11–20 ng/mL	27.46–49.92 nmol/L
Deficient	<11 ng/mL	<27.46 nmol/L

25(OH)D_3_ = 25-hydroxyvitamin D_3_; to convert from ng/mL to nmol/L, multiply by 2.496.

**Table 3:** Summary of studies in the relationships between vitamin D levels and aspects of osteoarthritis.

	Reference	Year	Country	Study design	Condition of samples	Samples	Age (years)^†^	Females (%)	Controls	Age (yrs)	% female	Vitamin D assay	Follow-up	Results
Development and progression	Lane et al. [11]	1999	USA	Case-cohort longitudinal	Participants of the Study of Osteoporotic Fractures (SOF)	237	>65	100	N/A	—	—	Radioimmunoassay	8 yrs	Low 25(OH)D = 3x as likely to develop hip OA (JSN) Low 25(OH)D ≠ osteophyte growth defined hip OA No association between 1,25(OH)_2_D_3_ and hip OA
	McAlindon et al. [12]	1996	USA	Prospective observational study	Participants of the Framingham Study	556	70.3 ± 4.5	—	N/A	—	—	Competitive protein-binding assay	8 yrs	Low intake and serum Vit D ≠ ↑ risk for progression of knee OA Vit D positively correlated with BMD, Vit D intake, and physical activity Vit D inversely correlated with BMI Low Vit D = increased risk of knee OA Vit D levels predict osteophyte growth and cartilage loss (less so)
	Konstari et al. [13]	2014	Finland	Longitudinal cohort	No hip or knee OA at baseline	5274	30–99	54.1	N/A	—	—		10 yrs	Low 25(OH)D ≠ development of hip or knee OA Baseline 25(OH)D was associated with known risk factors of OA except for traumatic injury
	Chaganti et al. [14]	2010	USA	Longitudinal cohort	Participants of the Osteoporotic Fractures in Men Study (MrOS)	1104	77.2 ± 5.3	0	N/A	—	—	MS	4.6 yrs	↑ 25(OH)D levels = ↓ prevalence of radiographic hip OA Vit D insufficient men (levels of 25[OH]D 15.1–30 ng/mL) = 2-fold ↑ likelihood of prevalent radiographic hip OA compared with Vit D sufficient men
	Abu El Maaty et al. [15]	2013	Egypt	Cross-sectional	Postmenopausal + clinically diagnosed knee OA	36	54.7 ± 3.2	100	10	25.8 ± 2	0	HPLC	N/A	↓ 25(OH)D levels are associated with newly diagnosed OA in postmenopausal Egyptian women
	Heidari et al. [16]	2011	Iran	Cross-sectional	Knee OA (ACR Criteria)	148	60.2 ± 12.9	—	150	60.1 ± 10.2	—	ELISA	N/A	↓ 25(OH)D in OA than control (NS) High prevalence rate of serum 25-OHD deficiency Significant positive association between serum 25(OH)D deficiency and knee OA in a subgroup of patients aged <60 years, greater association in younger patients
	Bergink et al. [17]	2009	Netherlands	Prospective population-based cohort study	Participants of the Rotterdam Study	1248	66.2 ± 6.7	58.3	N/A	—	—	Radioimmunoassay	6.5 yrs	↓ Dietary intake of vitamin D = ↑ knee ROA ↓ 25(OH)D = ↑ risk of progressive ROA, not in confounding factor adjusted results (including age, sex, BMD, smoking, JSN, fall tendency, health status, disability index, and caloric intake) ↓ Baseline BMD patients: ↓ 25(OH)D (serum and intake) = ↑ incidence of knee ROA
	Ding et al. [18]	2009	Australia	Cohort study, cross-sectional and longitudinal	Participants of the Tasmanian Older Adult Cohort Study (TASOAC); did not have RA	1002	51–79	50	N/A	—	—	Radioimmunoassay	2.9 yrs	Baseline Vit D insuff. = ↓ knee cartilage (medial and lateral tibial sites), regardless of sex, ROA status, and knee pain Baseline Vit D levels predicted changes in medial and lateral tibial cartilage volume ↑ Serum 25(OH)D levels = ↑ knee tibial cartilage volume in older people, particularly in females, ROA patients, and those with knee pain Vit D insuff. = moderate-to-severe JSN in older adults
	Felson et al. [19]	2007	USA	2 longitudinal cohort studies	Participants of the Framingham Study	715	Framingham: 53.1 ± 8.7	53.1	N/A	—	—	Radioimmunoassay	9.5 yrs	Baseline Vit D ≠ radiographic worsening ↑ Vit D = slightly ↑ rates of worsening (NS after adjustment for risk factors) Vit D def. = slight ↑ in risk of JSN (NS after adjustment for risk factors) ↓ Vit D = slight ↓ risk of osteophyte growth
						277	BOKS: 66.2 ± 9.3	41.4	N/A	—	—	Radioimmunoassay	30 mo	Baseline Vit D ≠ radiographic worsening ↑ Vit D = ↑ risk of worsening No Vit D def. = slight ↑ risk of JSN (NS after adjustment for risk factors) ↓ Vit D ≠ osteophyte growth Vit D def. appears slightly protective against cartilage loss

Pain and function	Muraki et al. [20]	2011	UK	Cross-sectional, cohort study	Participants of the Hertfordshire Cohort Study	787	65.6 ± 2.7	49.5	—	—	—	Chemiluminescent	—	25(OH)D ≠ knee ROA ↑ Vit D = ↑ knee pain Fok1 VDR polymorphism = knee ROA and pain
	Laslett et al. [21]	2014	Australia	Longitudinal population-based cohort study		769	62.1 ± 7.0	50.5	N/A	—	—	Radioimmunoassay	5 yrs	Vit D def. predicts knee pain over a 5-year period Vit D def. may predict hip pain over 2.4 yrs
	Yazmalar et al. [22]	2013	Turkey	Prospective cohort	Knee OA (ACR Criteria)	74	48.70 ± 7.14	67.6	70	41.39 ± 4.21	37	HPLC	~6 mo	No correlation between Vit D status and WOMAC or VAS
	Al-Jarallah et al. [23]	2011	Kuwait	Cross-sectional	Primary knee OA	99	56.49 ± 9.12	91	N/A	—	—	Radioimmunoassay	N/A	Most knee OA patients were Vit D def. 25(OH)D ≠ OA severity (radiographic) or functional assessment

BMD and body composition	Bischoff-Ferrari et al. [24]	2005	USA	Population-based cohort	Primary knee OA (Framingham Study)	228	74.4 ± 11.1	64	N/A	—	—	Radioimmunoassay and competitive protein-binding assay		↑ Vit D status = ↑ BMD (femoral neck) in primary knee ROA independent of sex, age, BMI, physical activity, knee pain, and disease severity
	Christensen et al. [25]	2012	Denmark	Prospective cohort study	Knee OA + obese (BMI >30 kg/m^2^)	175	62.6 ± 6.3	81	N/A	—	—	Microparticle chemiluminescence immunoassay	16 we	Knee OA patients on a formula diet had ↑ Vit D levels and BMD
	Hunter et al. [26]	2003	UK	Cross-sectional twin study	Participants of St. Thomas' UK Adult Twin Registry	1644	24–79	100	N/A	—	—	Radioimmunoassay	N/A	Knee OA patients have ↓ Vit D levels Vit D levels ≠ osteophytes
	Barker et al. [27]	2014	USA	Cross-sectional	Knee OA	56	48 ± 1	55	N/A	—	—	Chemiluminescent immunoassay	N/A	Vit D def. = quadriceps dysfunction but ≠ inflammatory cytokines

† means at baseline in longitudinal studies. Vit D: vitamin D; ROA: radiographic osteoarthritis; ACR: American College of Rheumatology; NS: not statistically significant; BMI: body mass index; BMD: bone mineral density; HPLC: high performance liquid chromatography; ELISA: enzyme-linked immunosorbent assay; MS: mass spectrometry; Insuff.: insufficient; Def.: deficient; ↓: lower/decreased; ↑: higher/increased; ≠: no association; =: association; yrs: years; mo: months; we: weeks; JSN: joints space narrowing; N/A: not applicable.

**Table 4:** Summaries of randomised clinical trials to assess the efficacy of vitamin D supplementation for the treatment of knee osteoarthritis.

Reference	Country	Supplement	Dose	Condition of samples	Vitamin D status of participants	Samples/placebo	Vitamin D assay	Follow-up	Results
Sanghi et al. [28] 2013	India	Cholecalciferol granules or placebo	60,000 IU per day for 10days followed by 60,000 IU once a month for 12 months	>40 yrs old ACR Criteria WOMAC pain >4 for at least 6 months 6 months of conventional treatment BMI <30 No previous fracture or surgery to knee	Vitamin D insufficiency (25(OH)D ≤ 50 nmol/L)	52/51	EIA	Multiple over a 12-month period	Vit D = ↓ VAS and WOMAC pain scores versus placebo Vit D = ↓ WOMAC physical + total versus placebo Vit D = ↑ serum calcium, 25(OH)D, and alkaline phosphatase versus placebo No difference in WOMAC stiffness

McAlindon et al. [29] 2013	USA	Cholecalciferol or placebo	2,000 IU daily with subsequent adjustment in 2000 IU increments at 4, 8, and 12 months for a target 25OHD level between 36 and 100 ng/mL	Age > 45 Symptomatic knee OA KL ≥ 2 (ACR Criteria) WOMAC = mild pain	Not selected for	73/73	LC/MS/MS	Multiple over a 24-month period	Vit D levels ↑ over the 2 years No significant difference in knee pain between groups No significant differences between cartilage loss, JSW, and BML size No significant difference in WOMAC pain or function scores

Vit D: vitamin D; BMI: body mass index; ↑: higher/increased; =: association; ACR: American College of Rheumatology; WOMAC: Western Ontario and McMaster Universities Osteoarthritis Index; KL: Kellgren-Lawrence grading; VAS: visual analogue score; JSW: joint space width; BML: bone marrow lesion; EIA: enzyme immunoassay; LC/MS/MS: liquid chromatography-tandem mass spectrometry.
